# The Photography and Memory Project: Adapting an Intergenerational Program During a Global Pandemic

**DOI:** 10.1093/geroni/igab046.1557

**Published:** 2021-12-17

**Authors:** Carson De Fries, Roddy MacInnes, Anne Walker, Amy DelPo, Leslie Hasche, Andrew Steward, Matthew Schilz

**Affiliations:** 1 University of Denver, Denver, Colorado, United States; 2 Denver Public Library, Denver, Colorado, United States; 3 University of Denver, Lone Tree, Colorado, United States

## Abstract

Since 2019, a university-community partnership has connected undergraduate students with older adults from independent living and community-based settings (i.e., library, art museum) for a photography-based intergenerational program. This study compares the implementation and impacts of this photography-based intergenerational program both in an in-person format before the COVID-19 pandemic (n=34) and an online format during the COVID-19 pandemic (n=25). Pre- and post- results from older adult and undergraduate student participants regarding social connection and evaluation comments from program participants and staff were compared between the pre-pandemic, in-person format, and the same program’s offering the next year in a virtual format. Results indicate that the benefits of this intergenerational photography program were experienced at a similar level during and before COVID-19. Findings demonstrate that intergenerational programs could effectively continue in remote formats, while also identifying potential challenges in implementation for staff regarding managing logistics and maintaining engagement among participants.

